# Ruminal microbiota and muscle metabolome characteristics of Tibetan plateau yaks fed different dietary protein levels

**DOI:** 10.3389/fmicb.2024.1275865

**Published:** 2024-02-14

**Authors:** Kaiyue Pang, Jianmei Wang, Shatuo Chai, Yingkui Yang, Xun Wang, Shujie Liu, Cheng Ding, ShuXiang Wang

**Affiliations:** ^1^Qinghai Academy of Animal Husbandry and Veterinary Sciences in Qinghai University, Xining, Qinghai, China; ^2^Key Laboratory of Plateau Grazing Animal Nutrition and Feed Science of Qinghai Province, Xining, Qinghai, China; ^3^Yak Engineering Technology Research Center of Qinghai Province, Xining, Qinghai, China; ^4^College of Veterinary Medicine, China Agricultural University, Beijing, China; ^5^Department of Agriculture and Rural Affairs, Zachen County, Shannan, Tibet Autonomous Region, Xizang, China

**Keywords:** yaks, protein levels, muscle, rumen, microbiota, metabolomics yaks, metabolomics

## Abstract

**Introduction:**

The dietary protein level plays a crucial role in maintaining the equilibrium of rumen microbiota in yaks. To explore the association between dietary protein levels, rumen microbiota, and muscle metabolites, we examined the rumen microbiome and muscle metabolome characteristics in yaks subjected to varying dietary protein levels.

**Methods:**

In this study, 36 yaks were randomly assigned to three groups (*n* = 12 per group): low dietary protein group (LP, 12% protein concentration), medium dietary protein group (MP, 14% protein concentration), and high dietary protein group (HP, 16% protein concentration).

**Results:**

16S rDNA sequencing revealed that the HP group exhibited the highest Chao1 and Observed_species indices, while the LP group demonstrated the lowest. Shannon and Simpson indices were significantly elevated in the MP group relative to the LP group (*P* < 0.05). At the genus level, the relative abundance of *Christensenellaceae_R-7_group* in the HP group was notably greater than that in the LP and MP groups (*P* < 0.05). Conversely, the relative abundance of *Rikenellaceae_RC9_gut_group* displayed an increasing tendency with escalating feed protein levels. Muscle metabolism analysis revealed that the content of the metabolite Uric acid was significantly higher in the LP group compared to the MP group (*P* < 0.05). The content of the metabolite L-(+)-Arabinose was significantly increased in the MP group compared to the HP group (*P* < 0.05), while the content of D-(-)-Glutamine and L-arginine was significantly reduced in the LP group (*P* < 0.05). The levels of metabolites 13-HPODE, Decanoylcarnitine, Lauric acid, L-(+)-Arabinose, and Uric acid were significantly elevated in the LP group relative to the HP group (*P* < 0.05). Furthermore, our observations disclosed correlations between rumen microbes and muscle metabolites. The relative abundance of *NK4A214_group* was negatively correlated with Orlistat concentration; the relative abundance of *Christensenellaceae_R-7_group* was positively correlated with D-(-)-Glutamine and L-arginine concentrations.

**Discussion:**

Our findings offer a foundation for comprehending the rumen microbiome of yaks subjected to different dietary protein levels and the intimately associated metabolic pathways of the yak muscle metabolome. Elucidating the rumen microbiome and muscle metabolome of yaks may facilitate the determination of dietary protein levels.

## 1 Introduction

The yak is an indigenous livestock species native to the Tibetan Plateau and serves as the primary means of production and livelihood for local herders (Long et al., 2008). Due to the seasonal temperature variations on the plateau, the low slaughter rate of yaks results in an imbalanced supply of yak meat. Yak housing and fattening technology have emerged to address this issue, and their promotion can help alleviate the problem of a disrupted seasonal supply of yak meat in the market, fostering yak industrialization. As the season has a limited impact on yak fattening, researchers are increasingly concentrating on enhancing and controlling yak feed rations.

Diet constitutes the material foundation for animals to maintain their vital activities and production, and its nutritional level directly influences animal growth and development (Katongole and Yan, 2020; Wang et al., 2020). The relationship between energy and protein ratios is a crucial indicator of nutritional levels, and a complex interrelationship exists between the two in ruminants. A sufficient protein level in the diet can supply necessary nutrients for rumen microorganisms. However, an excessive protein level in the diet may not only disrupt the rumen environment’s homeostasis, leading to changes in the abundance and diversity of rumen microorganisms, but also squander feed energy and protein, thereby increasing the ecological pressure on the environment. Thus, the judicious utilization of protein resources in feed can reduce feed waste due to overfeeding and decrease urinary nitrogen emissions and environmental pollution (Jonker et al., 1998; Luo et al., 2004; Hristov et al., 2011). Selecting the appropriate dietary protein level is essential for optimizing yak breeding.

The rumen is a complex microbial ecosystem, with rumen microbes playing a crucial role in the fermentation of plant proteins (Seshadri et al., 2018; Liu C. et al., 2019). Rumen microbiota is influenced by various factors, such as species, sex, and diet (Ma et al., 2019; Zhang et al., 2019; Zhao et al., 2019). The diet’s nutritional level is among the most critical factors that can alter the relative abundance of rumen flora (Park et al., 2020; Wang et al., 2021). Recent studies have reported that the number of bacteria in the rumen significantly increases with elevated dietary protein levels (Kim et al., 2014). da Silva-Marques et al. (2018) demonstrated that high protein level diets could enhance the relative abundance of ruminal Fibrobacter succinogenes and decrease that of Ruminococcus albus in Nellore cattle. However, the regulation of rumen microbiota in yaks by dietary protein levels remains unclear; thus, our study concentrates on this area.

Intramuscular fat (IMF) and its fatty acid composition are critical in determining the quality of meat consumed by humans (Hausman et al., 2009). Fat content and fatty acids in ruminants are primarily influenced by rumen diet nutrition and bacterial metabolism (Uccioni et al., 2012). Zhang et al. (2014) reported that supplementing high protein diets in early weaned yaks increased intramuscular fat accumulation. These results suggest that dietary protein concentration positively impacts growth performance, carcass characteristics, meat yield, and meat quality in animals. Muscle metabolites dictate the physiological and meat quality characteristics of muscles (Ritota et al., 2012; Muroya et al., 2020), and the properties of muscle metabolites can affect meat quality. Prior studies have demonstrated a link between hydrophilic amino acids, β-alanine, and the longissimus dorsi and intermuscular muscles in beef (Argyri et al., 2011; Muroya et al., 2014). Organic acids in beef have been shown to be an effective means of monitoring meat quality. The percentage of amino acid content in muscle significantly influences muscle nutritional value. Beef is regarded as a protein-rich food with a high concentration of easily absorbed essential amino acids in human daily nutrition due to its amino acid composition. However, there is limited information on the metabolomic biomarkers of dietary protein levels affecting muscle differences in yaks and on the mechanisms of improving muscle fatty acid distribution, amino acid composition, and other quality parameters in yaks fed different protein levels through rumen microbiota regulation. Consequently, we focused on the effects of dietary protein levels on muscle metabolic responses in yaks.

Currently, there are no established standards for the nutritional requirements of yaks, both domestically and internationally. Standard diets are typically formulated based on experience or by considering the nutritional requirements of beef cattle (National Research Council [NRC], 2007) when feeding yaks. Investigating appropriate protein levels in diets can provide a research foundation for ruminant studies and a theoretical basis for ruminant production, possessing high scientific and economic determining feeding standards for yaks. Nevertheless, the impact of dietary protein levels on the rumen microbiota and muscle metabolism of yaks, as well as the association between them, remains elusive. In the present study, we hypothesized that dietary protein levels influence the rumen microbiota and muscle metabolites of yaks, and that a potential correlation exists between yak rumen microbiota and muscle metabolome. Consequently, our study offers new insights into the interactions between rumen microbiota and muscle metabolome in yaks, which will aid in future diet configuration to affect rumen microbes and muscle metabolites to enhance yak growth performance.

## 2 Materials and methods

### 2.1 Experimental design

All experimental procedures and the handling of experimental animals were conducted in accordance with the guidelines of the Ethics Committee. The experiment was approved by the Animal Protection and Utilization Professional Committee of Qinghai University (approval number: QHU20200921) and was carried out at the standardized cattle and sheep breeding demonstration pasture in Haiyan Jinyintan, Qinghai Province, China. We initially selected 36 healthy 3-year-old male yaks with similar body weight (198.8 ± 18.7 kg) from grazing pastures and randomly divided them into three groups with 12 yaks each: low dietary protein group (LP, 12% protein concentration), medium dietary protein group (MP, 14% protein concentration), and high dietary protein group (HP, 16% protein concentration). The diets were formulated according to the Chinese Beef Cattle Feeding Standard (NY/T815-2004), and the diet composition and nutrient composition are shown in Table 1. All yaks were assigned unique identification numbers and housed in individual pens per group, with free access to water and fed twice a day at 8:00 and 17:00 with a total mixed ration. The acclimatization period was 15 days, followed by a regular feeding period of 270 days.

**TABLE 1:** Ingredient of the basal diet (DM basis).

Ingredients (%)	LP	MP	HP
Corn	23.80	16.70	22.00
Wheat	21.00	23.00	8.30
Wheat bran	4.50	1.90	4.50
Rapeseed meal	4.60	4.60	8.30
Soybean meal	7.20	14.50	18.10
5% Premix^1^	2.10	2.10	2.10
Calcium hydrogen phosphate	0.40	0.40	0.40
Sodium chloride	0.90	0.90	0.90
Limestone	0.60	0.60	0.60
Oat hay	17.10	10.00	10.00
Oat silage	11.80	21.70	21.20
Wheat straw	6.00	3.60	3.60
**Nutrient composition (%)**			
Crude protein	11.98	14.00	16.02
Metabolizable energy (MJ/kg)	10.66	10.66	10.66
Neutral detergent fiber	35.52	32.92	34.58
Acid detergent fiber	20.24	19.36	20.62
Calcium	0.36	0.38	0.40
Phosphorus	0.30	0.31	0.35

LP, low dietary protein concentration group; MP, medium dietary protein concentration group; HP, high dietary protein concentration group; DM, dry matter.

^1^Provided as per kilogram of premix: VA: 4000 IU, VD3 800 IU, VE 40 IU, Cu: 15 mg, Fe: 60 mg, Zn: 30 mg, Mn: 40 mg, Se: 0.3 mg, I: 0.8 mg, Co: 0.3 mg.

### 2.2 Ruminal fluid and muscle sampling and measurement

We selected the final 36 yaks for sampling. At the beginning and end of the trial period, the test yaks were weighed on an empty stomach before feeding, and the average daily weight gain and total weight gain were calculated. The number of feeds and residuals were recorded daily, and dry matter intake and F/G were calculated. At 3 to 4 h after feeding on the 90th day of the experiment. Crude protein (CP), neutral detergent fiber (NDF), acid detergent fiber (ADF), calcium (Ca), and phosphorus (P) were determined in each sample in the laboratory and ME was calculated. Mixed feeds (100 g) were collected and dried in a forced-air oven at 60°C for 48 h and then ground through a 1-mm sieve before analysis. CP, Ca and P contents were determined according to AOAC Procedure (1990). The NDF and ADF contents were determined by the method of Van Soest et al. (1991). All samples were collected on the same day, and each yak was sampled only once. Ruminal fluid was collected through a gastric tube sampler in the morning before feeding. The initial 100 mL of the collection was discarded and filtered through four layers of gauze, with the ruminal pH measured immediately using a bench top acidity meter (Model HI221, HANNA, Italy). The remaining ruminal fluid samples were dispensed into 15 mL centrifuge tubes, immediately frozen in liquid nitrogen, transported back to the laboratory, and stored at −80°C. Five rumen fluid samples from each group were randomly selected for 16S rDNA high-throughput sequencing. The 36 yaks were slaughtered according to GB/T 19477-2004 “Operating Procedures for Cattle Slaughter.” After the feeding period, the yaks were deprived of food and water before slaughter. A 200 mg sample of the longest muscle of the yak’s back (at the 12th–13th ribs) was taken after slaughter and stored in sterilized frozen tubes. Five muscle samples from each group were randomly selected for non-targeted metabolomics sequencing.

### 2.3 16S rDNA sequencing

Total genomic DNA was extracted from the samples using the CTAB method. PCR amplification of the extracted DNA was performed (pre-denaturation treatment at 94°C for 5 min, followed by a denaturation cycle at 94°C for 30 s, annealing at 50°C for 30 s, and extension at 72°C for 30 min, followed by extension at 72°C for 5 min). Different regions of the 16S rDNA gene (16S V3-V4) were amplified using primers 515F (5′-GTGCCAGCMGCCGCGG-3′) and 806R (5′-GTGCCAGCMGCCGCGG-3′). PCR was performed using a 25 μl amplification system, 5 μmol/L upstream and downstream primers, and approximately 5 ng of template DNA. PCR amplification products were detected by 1.0% agarose gel electrophoresis and purified using the MinElute Gel Extraction Kit (Qiagen, Germany). Library construction was performed with the TruSeq^®^ DNA PCR-Free Sample Preparation Kit (Illumina, USA), and the constructed libraries were quantified by Qubit and Q-PCR and sequenced on the Illumina NovaSeq6000 platform. Purification, library construction, and sequencing processes were performed by Novogene Bioinformatics (Beijing, China) for paired-end reads (2 × 250).

Sequences obtained from the Illumina NovaSeq6000 platform were processed through the open-source software pipeline QIIME (Quantitative Insights into Microbial Ecology) version 1.8.0-dev (Caporaso et al., 2010). Based on the barcode sequence information for each sample, sequences were first demultiplexed using an internal Perl script on the raw fastq file and subjected to the following criteria: on the 10 bp sliding window, the average mass fraction 20 at any site truncated 250 bp reads, discarding truncated reads shorter than 50 bp; discarding sequences for exact barcode matches, 2-nucleotide mismatches in primer matches, and readings containing ambiguous characters. The DADA2 method was used to filter, denoise, splice, and de-chimerize the sequencing data, perform clustering analysis on ASVs of raw data, and perform species difference analysis between sample groups based on ASVs and annotation results, among other analyses. Chimeric sequences were identified and removed using UCHIME (Edgar, 2010). The phylogenetic relationships of each 16S rDNA gene sequence (referred to as RSV in this paper) were analyzed using the UCLUST algorithm. Alpha diversity (Chao1, Shannon, PD-whole-tree and Observed-species) was calculated by MOTHUR (v1.30.2) based on QIIME 2 (version 1.9.0). Beta diversity was calculated using unweighted UniFracdistance and the results were visualized by Principal Coordinate Analysis (PCoA) and plotted against the GUniFrac and R software packages. To distinguish significant differences in abundance at the phylum and genus level of yak ruminal microbiota, we used the Statsp package in R and PYTHON and the Wilcoxon rank sum test in STAMP. All raw sequences (16S) were submitted to the NCBI Sequence Read Archive (SRA)^1^ after assembly and filtering, registration number SRP089832.

### 2.4 LC-MS untargeted metabonomics sequencing

After 100 mg of muscle tissue sample was ground with liquid nitrogen, 500 μL of an 80% methanol solution was added to mix evenly and placed in an EP tube. The mixture was shaken with a scroll oscillator and allowed to stand in a water bath for 5 min before centrifuging at 4°C for 20 min. After centrifugation, a certain volume of supernatant was taken and diluted with mass spectrometry water until the methanol content reached 53%. The sample was centrifuged again at 4°C for 20 min, and a certain amount of supernatant was collected and analyzed using a liquid chromatograph-mass spectrometer (LC-MS).

The LC-MS analyses were carried out with an Agilent 1290 Infinity LC system (Agilent Technologies, Santa Clara, CA, United States) with an Acquity BEH C18 column (100 mm × 2.1 mm i.d., 1.7 μm, Waters, Milford, MA, United States) preheated to 45°C. The mobile phase was composed of solvent A (aqueous 0.1% (v/v) formic acid) and solvent B (acetonitrile), delivered at a rate of 0.40 mL/min. The injection volume was 3 μL. The LTQ Orbitrap mass spectrometer (XL, Thermo Fisher Scientific, Waltham, MA, United States) was used to obtain sample mass spectrum data using positive or negative ion scan mode. The electron spray ionization source conditions were set as follows: sample voltage, 40 V; capillary voltage, 1.0 kV; ion source temperature, 120°C; desolvation gas rate and temperature, 900 L/h and 500°C. The mass range was 50–1000 m/z, and the scan time and interscan delay were 0.15 and 0.02 s, respectively. The normalized collision energy was 6 eV.

LECO’s Chroma TOF 4.3X software and the LECO Fiehn Rtx5 database (Kind et al., 2009) were used for raw peak extraction, data baseline filtering and baseline calibration, peak alignment, deconvolution analysis, peak identification, and peak area integration. A final data matrix containing retention times, mass-to-charge ratios (MZ), and peak intensities was obtained. To observe metabolic changes between groups, orthogonal partial least squares discriminant analysis (OPLS-DA) and principal component analysis (PCA) were performed using the R package model,^2^ and 7-fold cross-validation was used to assess model stability. Significantly different metabolites were screened based on the combination of significant predictor variables (VIP) and *t*-tests obtained from the OPLS-DA model. Significant predictor variables (VIP) values > 0.1 and *p*-values < 0.05 were considered as differential metabolites (DEMs) (Sreekumar et al., 2009; Haspel et al., 2014; Heischmann et al., 2016). log2(FC) >0 indicates up-regulation of metabolites, and log2(FC) <0 indicates down-regulation of metabolites. DEMs were identified through the Metabolome Database (BMDB) and the Kyoto Encyclopedia of Genes and Genomes (KEGG) for further identification and validation. Metabolic pathway distribution and metabolite set enrichment analysis using the MetaboAnalyst web server.^3^

### 2.5 Correlations between microbial communities and muscle metabolites

Differential muscle metabolites and significantly affected microflora were selected for further analysis using R (v3.2.4, see text footnote 2) to study changes in relevant metabolic processes. For Spearman correlation analysis, *P*-values were calculated using the Psych package (author, W. Revelle; publication date, 2016; version, 1.6.9),^4^ considering absolute Spearman correlations with *P* > 0.05 as significant. These correlations were visualized using the Pheatmap package (author, R. Kolde; publication date, 2015; version 1.0.8)^5^ in R and Cytoscape 2.8.2 (Smoot et al., 2011) for generating network visualizations.

### 2.6 Statistical analysis

Statistical analyses of data related to rumen microbial communities and muscle metabolomics were performed using SPSS version 20.0 (SPSS Inc., Chicago, IL, USA). One-way analysis of variance (ANOVA) and least significant difference (LSD) tests were employed to assess statistical differences, with data expressed as the mean and standard error of the mean (SEM). Differences were considered statistically significant when *P* < 0.05. Graphs were generated using GraphPad Prism version 7.00 (GraphPad Software, San Diego, CA, USA).

## 3 Results

### 3.1 Effects of diets with different protein levels on growth performance of yaks

Total weight gain in the MP group was higher than the other two groups, but the difference was not significant (*P* > 0.05), average daily weight gain of yaks in the MP group was significantly higher than that of the LP and HP groups (*P* < 0.05). Dry matter intake in the LP group was significantly higher than that of the HP group (*P* < 0.05), and the feed-to-weight ratio of the LP group was significantly higher than that of the MP and HP groups (*P* < 0.05) (Supplementary Table 1).

### 3.2 Analysis of variation in microbial community amplicon sequencing

**FIGURE 1:**
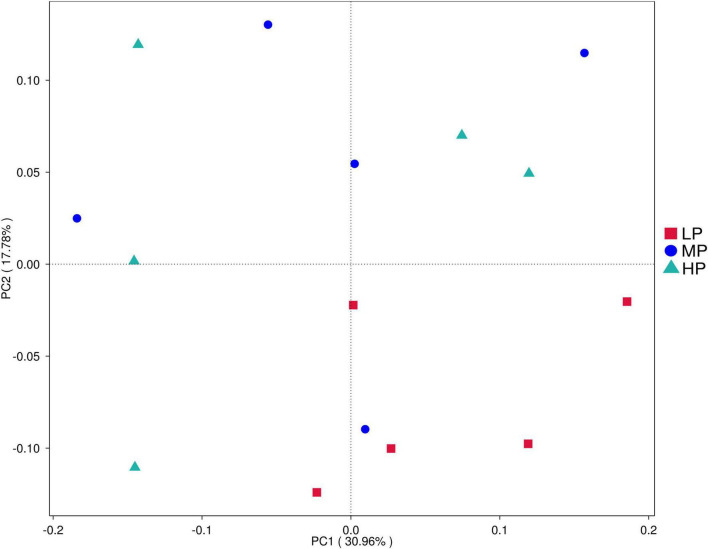
Principal component analysis (PCA) of rumen bacterial community. Yaks with low (LP), medium (MP), and high (HP) dietary protein concentrations.

To evaluate the differences in the rumen microbiota of yaks fed varying dietary protein levels, we conducted 16S rDNA sequencing. High-throughput Illumina sequencing technology was utilized to detect and characterize the entire rumen bacterial composition by targeting the V3 and V4 regions of 16S rDNA. A total of 1,229,150 reads were obtained from the Illumina MiSeq platform sequencing run of 15 rumen fluid samples, averaging 81,943 sequences per sample. After quality control, there were 814,104 clean sequences with an average of 54,273 clean sequences per sample. Clean sequences from the 15 rumen fluid samples were clustered based on their ASVs, resulting in a total of 7,748 ASVs. Alpha diversity was measured within the sample based on the results of species annotation ASVs. Principal coordinate analysis (Figure 1) demonstrated separation between the LP group, MP group, and HP group, indicating that differences in dietary protein levels were a key factor in these distinctions. In rumen fluid samples, Chao 1 values and observed_otus indices were higher in the HP group than in the MP and LP groups. Shannon indices were significantly higher in the HP and MP groups (*P* < 0.05), while Simpson indices were significantly higher in the MP group compared to the LP group (*P* < 0.05) (Table 2).

**TABLE 2:** The alpha diversity index of rumen bacteria in yaks with different dietary protein concentrations.

Item	LP	MP	HP	SEM	*P*-value
Chao 1	1129.762	1260.323	1290.879	33.42	0.106
Observed_ species	1126.400	1257.200	1289.200	33.26	0.099
Shannon	8.489^b^	9.153^a^	8.987^a^	0.11	0.018
Simpson	0.987^b^	0.995^a^	0.993^ab^	0.01	0.041

LP, Low dietary protein concentration group; MP, medium dietary protein concentration group; HP, high dietary protein concentration group. The data are expressed as means, different superscript letters in the same row indicate significant differences between groups (*P* < 0.05).

**FIGURE 2:**
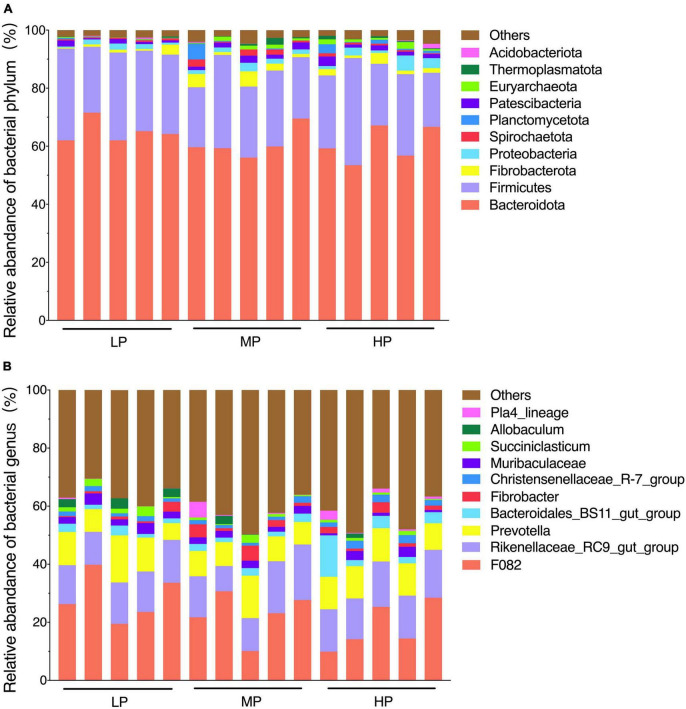
Rumen bacterial community composition. The dominant phylum **(A)** (top 10 relative abundance); genus **(B)** (top 10 relative abundance). Yaks with low (LP), medium (MP) and high (HP) dietary protein concentrations.

**FIGURE 3:**
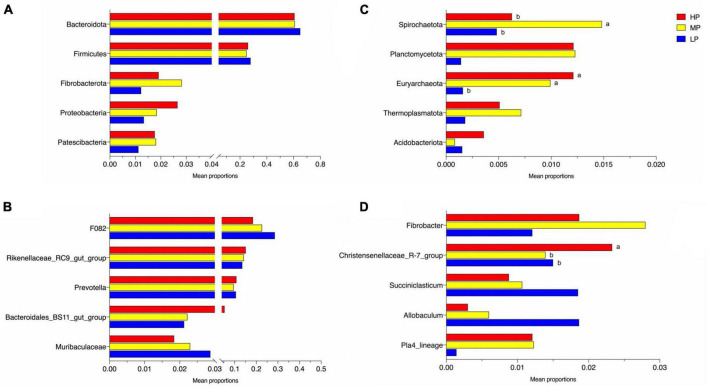
Significantly altered relative abundance of rumen bacteria of phylum **(A,B)** and genus **(C,D)**. Different letters of ab in the graph indicate significantly different values (*P* < 0.05). Yaks with low (LP), medium (MP), and high (HP) dietary protein concentrations.

The total relative abundance of Bacteroidota and Firmicutes was 62.21 and 26.27% in the LP, MP, and HP groups, respectively, and these were identified as the significant bacterial phyla based on their assignment. The less abundant phyla included Fibrobacterota, Proteobacteria, Patescibacteria, Spirochaetota, Planctomycetota, Euryarchaeota, Thermoplasmatota, and Acidobacteriota (Figure 2A). As expected, we observed significant changes in bacterial phyla in the rumen of yaks fed different feed protein levels (Figures 3A, B). For the relative abundance of Bacteroidota and Firmicutes in the rumen microbiota, we observed a decreasing trend in the relative abundance of Bacteroidota with increasing feed protein levels. The relative abundance of Firmicutes was higher in the LP group and lower in the MP group. Notably, the relative abundance of Bacteroidota and Firmicutes did not differ significantly among the LP, MP, and HP groups. The relative abundance of Spirochaetota was significantly higher in the MP group compared to other feed protein levels (*P* < 0.05). At the same time, there was no significant difference between the LP and HP groups. In addition, the relative abundance of Euryarchaeota was significantly higher in the MP and HP groups than in the LP group (*P* < 0.05). At the same time, there was no significant difference between the MP and HP groups.

The detected ASV sequences were assigned to 568 genera. In total, 16 of these genera represented more than 1% of all samples at different feed protein levels, with *F082* (23.24%), *Rikenellaceae_RC9_gut_group* (14.29%), and *Prevotella* (10.32%) being the most dominant genera, followed by *Bacteroidales_BS11_gut_group* (3.22%), *Muribaculaceae* (2.34%), *Fibrobacter* (1.96%), *Christensenellaceae_R-7_group* (1.74%), *UCG-010* (1.68%), *Eubacterium_coprostanoligenes_group* (1.64%), and *Papillibacter* (1.58%) (Figure 2B). As shown in Figures 3C, D, the relative abundance of *Christensenellaceae_R-7_group* in the HP group was significantly higher than that of LP and MP groups (*P* < 0.05), while with increasing feed protein levels *Rikenellaceae_RC9_gut_group*. The relative abundance of *Prevotella* was highest in the HP group and lowest in the MP group.

### 3.3 Metabolite differences in the muscle of yaks with different dietary intake protein levels

**FIGURE 4:**
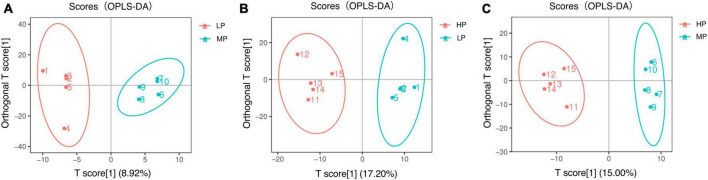
Orthogonal partial least squares discriminant analysis (OPLS-DA) scores for two-by-two comparison of metabolomics. Yaks with low (LP), medium (MP), and high (HP) dietary protein concentrations.

**FIGURE 5:**
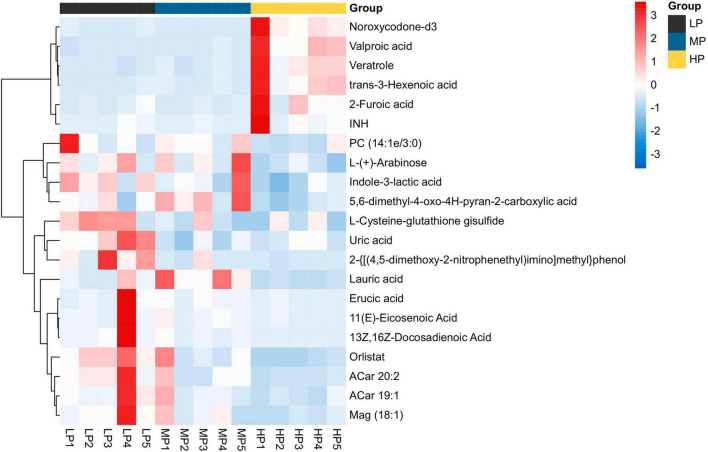
Heat map analysis of differential metabolite hierarchical clustering analysis for identifying VIP > 1 in muscle metabolism groups of yaks in LP, MP, and HP groups. Black squares represent the LP group, blue squares represent the MP group, and yellow squares represent the HP group. Each row represents one metabolite; each column represents one sample. The coloring is based on the signal intensity measured by LC-MS. Red represents high signal intensity, blue represents low signal intensity, and white cells represent intermediate (see the color scale on the right side of the heat map).

**FIGURE 6:**
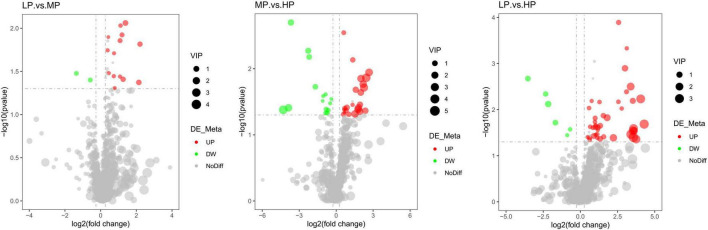
Volcano plot of differential metabolites for a two-by-two comparison (red represents up-regulated metabolites and green represents down-regulated metabolites). Yaks with low (LP), medium (MP), and high (HP) dietary protein concentrations.

**FIGURE 7:**
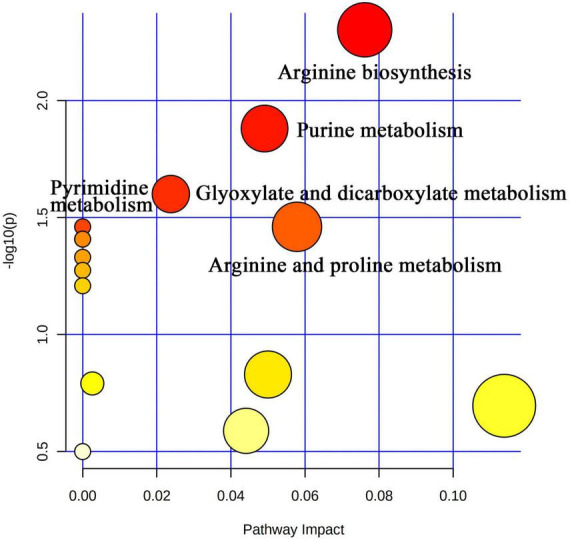
Visual analysis of metabolic pathways by MetPA software with bubble plots. The size of the bubbles is proportional to the effect of each channel; bubble color represents significance, with red being the highest and white the lowest; *P*-values less than 0.05 and pathway impact factors greater than 0.5 indicate that the pathway is more affected.

Liquid chromatograph-MS analysis of muscle metabolites from yaks in the LP, MP, and HP groups identified a total of 786 metabolites. As illustrated in the figure, pairwise comparisons of the three groups, along with orthogonal partial least squares discriminant analysis (OPLS-DA), demonstrated a primary unsupervised separation among the groups (Figure 4). The score plots revealed significant separation between the LP and MP groups, the MP and HP groups, and the LP and HP groups. Subsequently, we further examined these three datasets to identify differential metabolites between the groups. A total of 16 differential metabolites were found between the LP and MP groups, with 14 exhibiting significantly upregulated levels and 2 downregulated; 38 differential metabolites were observed between the MP and HP groups, with 23 displaying significantly upregulated levels and 15 downregulated; 46 distinct metabolites were detected between the LP and HP groups, with 40 showing considerably upregulated differential metabolite levels and 6 downregulated metabolites (VIP > 0.1, *P* < 0.05) (Figure 5). Ultimately, Figure 6 highlights 21 differential metabolites with variable importance predictions greater than 1.0. In general, a comparison among the three treatment groups identified 34 differential metabolites (Table 3). Notably, these primary differential metabolites mainly comprised lipids and lipid-like molecules (11 metabolites), organic acids and derivatives (7 metabolites), organic oxygen compounds (5 metabolites), organoheterocyclic compounds (5 metabolites), benzenoids (3 metabolites), coumarins and derivatives (1 metabolite), and organic nitrogen compounds (1 metabolite). Among these, in the LP and MP groups, the content of metabolites 2-hydroxy-2-methylbutanoic acid, 4-methylvaleric acid, tetrahydrocortisone, L-cysteine-glutathione gisulfide, 4-acetamidobutyric acid, N-acetylglucosamine 1-phosphate, uric acid, and esculin in the LP group was significantly higher than in the MP group, whereas the content of the metabolite D-mannitol 1-phosphate was notably lower. In the MP and HP groups, the contents of 2-ethylhexanoic acid, lysopc 16:1, D-erythrose 4-phosphate, and L-(+)-arabinose in the MP group increased significantly compared to those in the HP group, while the contents of D-(-)-glutamine, 4-acetamidobutyric acid, L-arginine, *trans-*3-hexenoic acid, D-(-)-mannitol, thymine, and lipoic acid decreased significantly. In the LP and HP groups, LP group metabolites 2-ethylhexanoic acid, 3-methylglutaric acid, 13-HPODE, decanoylcarnitine, lauric acid, L-cysteine-glutathione gisulfide, orlistat, L-(+)-arabinose, uric acid, xanthine, indole-3-lactic acid, o-toluic acid, monobutyl phthalate, esculin, and N, N-dimethyldecylamine N-oxide concentrations increased significantly compared to those in the HP group. The contents of prostaglandin A2, valproic acid, *trans-*3-hexenoic acid, *cis-*aconitic acid, and veratrole decreased significantly. Based on the analysis of key metabolic pathways in the positive and negative ion modes of the KEGG database, metabolic pathways with a *P* < 0.05 were selected as the main influential pathways, as shown in Table 4. The 34 differential metabolites were involved in a total of 8 critical metabolic pathways: Arginine biosynthesis, Purine metabolism, Glyoxylate and dicarboxylate metabolism, Pyrimidine metabolism, Arginine and proline metabolism, D-Glutamine and D-glutamate metabolism, and Linoleic acid metabolism. These pathways are primarily involved in amino acid metabolism and energy metabolism (Figure 7). Additionally, Arginine biosynthesis and Purine metabolism exhibited higher effect values, suggesting these pathways are more essential.

**TABLE 3:** Significant changes in muscle metabolites of yaks at different protein levels.

Biological roles	Metabolite name	LP vs. MP	MP vs. HP	LP vs. HP	Rtmed^3^	Mzmed^4^	Chemical structure
Lipids and lipid-like molecules	2-Hydroxy-2-methylbutanoic acid	↑ Up^1^	None	None	5.11	117.056	C5 H10 O3
	4-Methylvaleric Acid	↑ Up	None	None	5.415	115.077	C6 H12 O2
	Tetrahydrocortisone	↑ Up	None	None	5.962	382.258	C21 H32 O5
	2-Ethylhexanoic acid	None	↑ Up	↑ Up	5.734	143.108	C8 H16 O2
	Lysopc 16:1	None	↑ Up	None	8.875	492.310	C24 H48 N O7 P
	3-Methylglutaric acid	None	None	↑ Up	3.208	145.051	C6 H10 O4
	13-HPODE	None	None	↑ Up	6.235	313.237	C18 H32 O4
	Prostaglandin A2	None	None	↓ Down^2^	7.304	333.208	C20 H30 O4
	Valproic acid	None	None	↓ Down	5.823	145.122	C8 H16 O2
	Decanoylcarnitine	None	None	↑ Up	6.052	316.248	C17 H33 N O4
	Lauric acid	None	None	↑ Up	6.427	199.171	C12 H24 O2
Organic acids and derivatives	L-Cysteine-glutathione gisulfide	↑ Up	None	↑ Up	1.367	425.081	C13 H22 N4 O8 S2
	D-(-)-Glutamine	None	↓ Down	None	1.347	145.062	C5 H10 N2 O3
	4-Acetamidobutyric Acid	↑ Up	↓ Down	None	1.351	144.067	C6 H11 N O3
	L-arginine	None	↓ Down	None	1.222	173.105	C6 H14 N4 O2
	*Trans-*3-Hexenoic acid	None	↓ Down	↓ Down	5.048	115.075	C6 H10 O2
	*Cis-*Aconitic acid	None	None	↓ Down	2.08	173.009	C6 H6 O6
	Orlistat	None	None	↑ Up	7.869	496.399	C29 H53 N O5
Organic oxygen compounds	D-Mannitol 1-phosphate	↓ Down	None	None	1.518	261.043	C6 H15 O9 P
	N-Acetylglucosamine 1-phosphate	↑ Up	None	None	1.072	300.049	C8 H16 N O9 P
	D-(-)-Mannitol	None	↓ Down	None	1.296	181.072	C6 H14 O6
	D-Erythrose 4-phosphate	None	↑ Up	None	1.200	199.002	C4 H9 O7 P
	L-(+)-Arabinose	None	↑ Up	↑ Up	1.381	149.046	C5 H10 O5
Organoheterocyclic compounds	Uric acid	↑ Up	None	↑ Up	1.806	167.021	C5 H4 N4 O3
	Thymine	None	↓ Down	None	1.269	125.036	C5 H6 N2 O2
	Lipoic acid	None	↓ Down	None	2.219	205.036	C8 H14 O2 S2
	Xanthine	None	None	↑ Up	2.117	151.026	C5 H4 N4 O2
	Indole-3-lactic acid	None	None	↑ Up	5.568	206.081	C11 H11 N O3
Benzenoids	o-Toluic Acid	None	None	↑ Up	5.173	135.046	C8 H8 O2
	Monobutyl phthalate	None	None	↑ Up	6.21	221.082	C12 H14 O4
	Veratrole	None	None	↓ Down	5.196	139.073	C8 H10 O2
Coumarins and derivatives	Esculin	↑ Up	None	↑ Up	5.172	339.069	C15 H16 O9
Organic nitrogen compounds	N,N-Dimethyldecylamine N-oxide	None	None	↑ Up	5.954	202.216	C12 H27 N O

^1^The“↓” means that the relative peak area of metabolites in the LP group was significantly lower compared with the HP or MP group the relative peak area of metabolites in the MP group was significantly lower compared with the HP group. “None” means that there is no significant difference between the LP group and MP group, between the MP group and the HP group, and between the LP group and the HP group.

^2^“Down” and “Up” indicate *P* < 0.05, which is adjusted by Bonferroni’s correction.

^3^Rtmed, median of m/z.

^4^Mzmed, the median of retention time.

**TABLE 4:** The results of metabolic pathways influenced the detection of important metabolites between groups based on MetaboAnalyst 3.0.

Metabolite pathway name	Total compounds^1^	Hits^2^	Raw *P*-Value^3^	Impact
Arginine biosynthesis	14	2	0.005	0.076
Purine metabolism	66	3	0.013	0.049
Glyoxylate and dicarboxylate metabolism	32	2	0.025	0.024
Pyrimidine metabolism	38	2	0.035	0.000
Arginine and proline metabolism	38	2	0.035	0.058
D-Glutamine and D-glutamate metabolism	5	1	0.039	0.000
Linoleic acid metabolism	5	1	0.039	0.000
Nitrogen metabolism	6	1	0.047	0.000

^1^Total compounds indicate the number of compounds in the pathway.

^2^The Hits indicate the actual matched number from the user-uploaded data.

^3^The Raw *P*-value is the original *P*-value calculated from enrichment analysis.

### 3.4 Correlation between muscle metabolome and rumen microbiome

**FIGURE 8:**
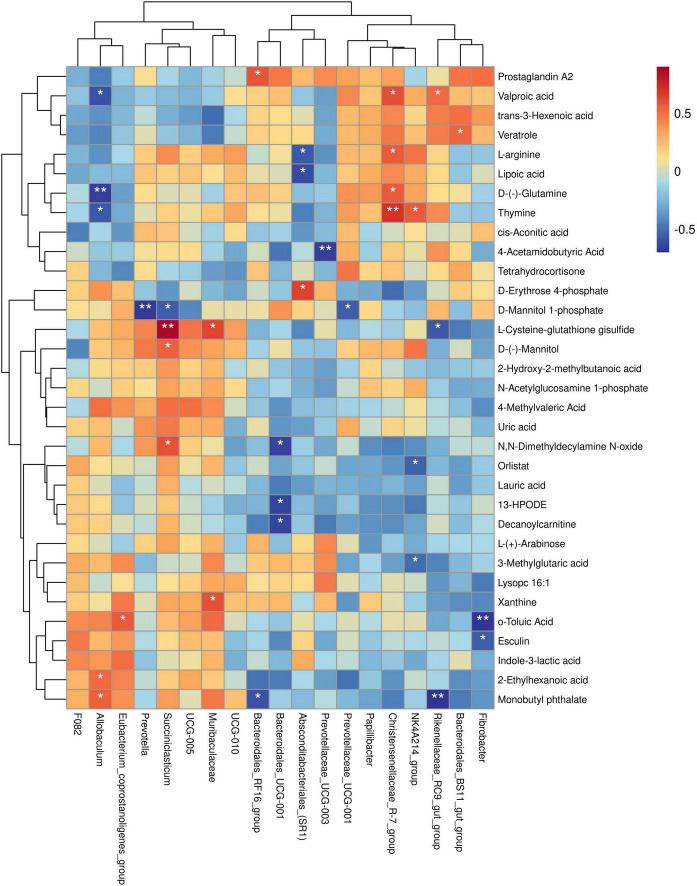
Spearman’s correlation study between yak rumen bacterial genera and muscle differential metabolites. Each row in the figure represents a metabolite, each column represents a genus, and the color of each cell represents the Spearman correlation coefficient between metabolite and genus levels (red indicates positive correlation, blue indicates negative correlation). *P < 0.05, **P < 0.01.

To further investigate the relationship between rumen bacterial genera and muscle differential metabolites, we conducted a correlation analysis between the relative abundance of specific rumen bacterial genera and the concentrations of muscle differential metabolites. The findings of this study revealed the following associations: The relative abundance of *Fibrobacter* was negatively correlated with the concentrations of o-toluic Acid and Esculin. The relative abundance of *Bacteroidales_BS11_gut_group* was positively correlated with the concentration of *Veratrole*. The relative abundance of *Rikenellaceae_RC9_gut_group* was negatively correlated with L-Cysteine-glutathione disulfide and Monobutyl phthalate concentrations, and positively correlated with Valproic acid concentration. The relative abundance of *NK4A214_group* was negatively correlated with 3-Methylglutaric acid and Orlistat concentration, and positively correlated with Thymine concentration. The relative abundance of *Christensenellaceae_R-7_group* was positively correlated with Valproic acid, D-(-)-Glutamine, L-arginine, and Thymine concentrations. The relative abundance of *UCG-001* was negatively correlated with the concentration of D-Mannitol 1-phosphate. The relative abundance of *Prevotellaceae_UCG-003* was negatively correlated with the concentration of 4-Acetamidobutyric Acid. The relative abundance of *Absconditabacteriales_(SR1)* was negatively correlated with the concentrations of L-arginine and Lipoic acid, and positively correlated with the concentration of D-Erythrose 4-phosphate. The relative abundance of *Bacteroidales_UCG-001* was negatively correlated with the concentrations of 13-HPODE, Decanoylcarnitine, and N,N-Dimethyldecylamine N-oxide. The relative abundance of *Bacteroidales_RF16_group* was negatively correlated with the concentration of Monobutyl phthalate and positively correlated with the concentration of Prostaglandin A2. The relative abundance of *Muribaculaceae* was positively correlated with L-Cysteine-glutathione disulfide and Xanthine concentrations. The relative abundance of *Succiniclasticum* was negatively correlated with D-Mannitol 1-phosphate concentration and positively correlated with L-Cysteine-glutathione disulfide, D-(-)-Mannitol, and N,N-Dimethyldecylamine N-oxide concentrations. The relative abundance of Prevotella was negatively correlated with the concentration of D-Mannitol 1-phosphate. The relative abundance of *Eubacterium_coprostanoligenes_group* was positively correlated with o-Toluic Acid concentration. The relative abundance of *Allobaculum* was negatively correlated with Valproic acid, D-(-)-Glutamine, and Thymine concentrations, and positively correlated with 2-Ethylhexanoic acid and Monobutyl phthalate concentrations (*P* < 0.05) (Figure 8).

## 4 Discussion

In this study, we examined the rumen microbiome and muscle metabolome of yaks fed different dietary protein levels. Our findings shed light on the characteristics of the rumen microbiome and muscle metabolome in yaks subjected to varying dietary protein levels, and establish the relationship between the composition and function of the rumen microbiome and muscle metabolism. The microbiome is closely related to dietary regulation (Million et al., 2012; Evans et al., 2013), and dietary protein levels play a crucial role in the growth and reproduction of ruminants. Optimal dietary protein levels can enhance yak growth (Zhang et al., 2014), which may provide the foundation for an effective strategy to differentiate between low-protein (LP), medium-protein (MP), and high-protein (HP) groups of yaks. We assessed the rumen microbiota composition of yaks under different dietary protein levels by utilizing high-throughput Illumina sequencing of the 16S rDNA V3-V4 region, and employed LC-MS to characterize the yak muscle metabolome. Analyzing the

variations in the rumen microbiome can help elucidate the impact of different dietary protein levels on yaks. Firstly, our study found significant differences in both alpha and beta diversity indices of the microbiota among the LP, MP, and HP groups, suggesting that the diversity of rumen microbiota in yaks is closely related to dietary protein levels. The highest Chao1 and Observed_species indices were observed in the HP group, while the lowest were in the LP group, indicating that the richness of the rumen flora in yaks increased gradually with rising dietary protein levels. Both Shannon and Simpson indices were significantly higher in the MP group than in the LP group, suggesting that elevated dietary protein levels may support greater bacterial diversity. However, extremely high or low bacterial diversity may not necessarily promote a healthy rumen environment (Latham et al., 2018). Beta diversity displayed distinct clustering but also exhibited overlap among the LP, MP, and HP groups, indicating that these three groups share similar rumen microbiota.

In the current study, Firmicutes and Bacteroidota were the dominant phyla of rumen microorganisms in all three groups of yaks, consistent with previous studies (Zhou et al., 2017; Bi et al., 2018). Prior research has demonstrated that protein levels, a significant factor in ruminant diet formulation, have a substantial impact on the dominant rumen flora (Cui et al., 2019). Firmicutes degrade organic matter such as cellulose, protein, polysaccharides, and amino acids through metabolism (Levén et al., 2007). Bacteroidota break down large molecules of dissolved organic carbon such as proteins and polysaccharides (Spirito et al., 2018), and their higher proportions give them an advantage in nutrient utilization, which is essential for ruminants nutritional metabolism. We observed that the relative abundance of Firmicutes and Bacteroidota in the rumen of yaks in the LP group was higher than those in the MP and HP groups. This suggests that the relative abundance of rumen Firmicutes and Bacteroidota is higher for lower dietary protein levels, meaning that high-nutrient diets reduce their relative abundance, in agreement with the study by Ley et al. (2006). Proteobacteria, a typical marker of gastrointestinal flora imbalance (Pham et al., 2019), were significantly reduced in the LP group, presumably due to increased production of short-chain fatty acids and lactic acid, which lowered rumen pH and inhibited the growth of harmful rumen bacteria (Chen et al., 2016). This supports the notion that a 12% protein level can improve the ecological balance of yak rumen flora. Spirochaetota has been reported to play an important role in the hydrolysis of complex polysaccharides as well as the degradation of B vitamins and proteins in the rumen (Hernández et al., 2022). In the present study, we found that the relative abundance of Spirochaetota in the MP group was significantly higher than that in the LP and HP groups. Additionally, the average daily weight gain of yaks in the MP group was significantly higher than that of the LP and HP groups. This disparity can be attributed to the positive correlation between dietary protein levels and the apparent digestibility of crude protein and neutral detergent fiber (Van Dung et al., 2013). The enhanced digestibility of these nutrients resulted in an increased availability of energy and essential nutrients for the organism’s growth. Consequently, these findings suggest that yaks fed a protein level of 14% have a higher efficiency in the conversion and utilization of feed. Some spectra of Euryarchaeota have roles in metabolizing methane and degrading certain hydrocarbons (Baker et al., 2020) and play a role in yak feed utilization and methane emissions, among others (Zhou et al., 2017). In addition, methane emissions from yaks are usually lower than other breeds of cattle due to Euryarchaeota (Sha et al., 2020). In this study, we found that the relative abundance of rumen Euryarchaeota was significantly higher in the MP and HP groups than in the LP group, suggesting that yaks fed 14 and 16% protein levels may have higher energy utilization efficiencies, and also suggesting that yak rumen bacteria have the ability to adapt in structural composition in response to changes in diet. At the genus level, F082, *Rikenellaceae_RC9_gut_group*, and *Prevotella* were the dominant bacteria in the rumen. *Prevotella* is consistently dominant in the rumen, regardless of diet composition (Bi et al., 2018; Sun et al., 2019). The *Rikenellaceae_RC9_gut_group* belongs to the *Rikenellaceae* family, and most members of this group can ferment unabsorbed polysaccharides in the host intestine, producing short-chain fatty acids (SCFAs) such as acetic acid, propionic acid, and butyric acid (Su et al., 2014). *Rikenellaceae_RC9_gut_group* confirms its essential role in protein fermentation with increasing dietary protein levels (Yang et al., 2020). *Prevotella* is the predominant protein-degrading bacterium in the rumen, breaking down starch and some cell wall polysaccharides (Fernando et al., 2010). *Prevotella* utilizes protein, starch, and hemicellulose to generate various end products (Stevenson and Weimer, 2007; Carberry et al., 2012). Ellison et al. (2017) found that differences in crude protein levels, neutral detergent fiber, and acidic detergent fiber in the diet caused differences in the relative abundance of *Prevotella* in the rumen of ruminants. In our study, we observed a lower abundance of *Prevotella* in the MP group than in the LP and HP groups. The results of this study may be related to the particular breed of yak and the composition of the diet, and the highest abundance of *Prevotella* was observed in the HP group, indicating that the high protein diet promoted the proliferation of *Prevotella*. Additionally, Christensenellaceae are widely present in the gastrointestinal tract and mucosa of animals and are closely associated with high protein and fiber diets and fat deposition (Waters and Ley, 2019). *Christensenellaceae_R_7_group* plays an essential role in maintaining the structure and function of the gastrointestinal tract and in immune regulation within the animal organism (He et al., 2019; Liu J. et al., 2019). In our study, we found that the abundance of *Christensenellaceae_R_7_group* was significantly higher in the HP group than in the LP and MP groups. We speculate that *Christensenellaceae_R_7_group* is more likely to proliferate under high protein level diets. High protein diets may provide more fermentation and energy substrates for *Christensenellaceae_R_7_group*, and the increased microbial population in turn improves the efficiency of partial nutrient degradation (Singh et al., 2012).

Ruminal microbiota exhibit differences in their functions and metabolic pathways, which ultimately determine the production of fermentation end products and their impact on the host animal (Hungate, 1966). In this study, we first investigated the muscle metabolome of yaks fed different dietary protein levels. The results of OPLS-DA illustrate that there are significant differences in the composition of muscle metabolites among the three groups of yaks. Significant differences were found in Arginine biosynthesis, Purine metabolism, Glyoxylate and dicarboxylate metabolism, Pyrimidine metabolism, Arginine and proline metabolism, D-Glutamine and D-glutamate metabolism, Linoleic acid metabolism, and Nitrogen metabolism-related pathways between the three groups. Uric acid, the final metabolite of purines, is closely associated with TG, HDL, and fatty liver (Keenan et al., 2012) and is considered the most significant factor related to health status (Sampa et al., 2020). Several studies have found a correlation between body mass index (BMI) and uric acid, with fat content being associated with individual BMI (Chen et al., 2021). Dai et al. (2013) observed a strong association between increased BMI and elevated uric acid levels. Our study showed that the LP group had higher uric acid content, indicating that yaks fed a 12 percent protein level diet had higher body fat content. L-(+)-Arabinose, a component of hemicellulose, was found in lower levels in yaks in the HP group, suggesting that yaks in this group rapidly used these degradation products to meet their energy requirements based on the higher abundance of hemicellulose-degrading bacteria. 13-HPODE, a linoleic acid derivative detected by LC-MS (El Khoury et al., 2020), is an essential fatty acid for animal nutrition (Marangoni et al., 2020; Hamilton and Klett, 2021). Linoleic acid not only provides energy for vital activities and plays a crucial role in lipogenesis (Shao et al., 2020) but also has physiological effects such as regulating lipid metabolism, promoting growth and development, and improving organism immunity. Our study showed that yaks in the LP group had higher levels of 13-HPODE, suggesting that higher utilization of 13-HPODE by yaks consuming diets with 12% protein level may promote lipogenesis. We also detected organic acids and derivatives, such as L-arginine and D-(-)-Glutamine. Arginine is a conditionally essential amino acid that is an important regulator of growth, reproduction, and homeostatic metabolic pathways in animals (Morris Sidney, 2009). Arginine improves meat color stability, presumably related to its ability to delay mitochondria-mediated apoptosis (Tuell et al., 2021). Glutamine is an abundant free amino acid in animal plasma. It is an important precursor for protein, nucleotide, and amino sugar synthesis in animals and plays a vital role in maintaining acid-base balance, regulating immune function, and providing raw materials for energy metabolism (Souba, 1993; Wu et al., 1995). We found that the relative abundance of L-arginine and D-(-)-Glutamine increased with increasing dietary protein levels, suggesting that increasing dietary protein levels may contribute to improving yak meat quality and antioxidant capacity. Decanoylcarnitine is an important metabolite involved in energy metabolism and is an acylcarnitine. Carnitine combines with fatty acids or deamination products of branched-chain amino acids to form acylcarnitine, which transports metabolites through the cytoplasm to the mitochondria, where β-oxidation occurs during degradation to produce energy (Yang et al., 2021) and has the function of promoting lipid metabolism (Maruyama et al., 2017). Lauric acid is a specific saturated fatty acid with the longest carbon chain among medium-chain fatty acids and does not have cardiovascular disease risk. Lauric acid was previously found to be an essential substance in regulating energy metabolism in animal studies and improves coagulation factors and the antioxidant status of the body (Thijssen and Mensink, 2005). Our study showed higher levels of Decanoylcarnitine and Lauric acid in yaks fed on 12% protein level diets, suggesting that feeding 12% protein level diets may facilitate higher energy and fat deposition in yaks. When fat is deposited to a certain extent, it can improve the quality and flavor of yaks beef. Finally, KEGG pathway enrichment analysis showed that there were significant differences in Arginine biosynthesis, Purine metabolism, Glyoxylate and dicarboxylate metabolism, Pyrimidine metabolism, Arginine and proline metabolism, D-Glutamine and D-glutamate metabolism, Linoleic acid metabolism, and Nitrogen metabolism-related pathways between the three groups. Therefore, the yak muscle metabolomics indicated that the activation pathways of muscle metabolites in response to dietary nutrition were different in yaks with different dietary protein concentrations.

The significant correlation between rumen microbes and muscle metabolites reveals a potential relationship between these bacteria and muscle metabolites. *NK4A214_group* is a Ruminaceae-associated bacterium involved in the degradation of cellulose, providing an energy source for the host (Biddle et al., 2013). A recent study showed a positive correlation between the *NK4A214_group* and concentrations of Isobutyrate and Isovalerate (Liu C. et al., 2019). Ladeira et al. (2016) found that ruminal propionic acid production could increase intramuscular fat deposition in beef cattle. Orlistat is a gastrointestinal lipase inhibitor (Al-Nada, 2020) that impedes the digestion and absorption of lipid-rich foods, decreases food conversion, and leads to a compensatory increase in animal intake to reduce the systemic absorption of dietary fat (Hvizdos and Markham, 1999). In our study, the relative abundance of *NK4A214_group* was negatively correlated with orlistat concentration, leading us to conjecture that the rumen microorganism *NK4A214_group* in yaks provides more energy and increases muscle fat deposition by elevating its relative abundance. The decrease in orlistat concentration ensures healthy growth and muscle fat deposition in yaks. The relative abundance of *Christensenellaceae_R-7_group* in this study was positively correlated with the concentrations of D-(-)-Glutamine and L-arginine. This can be explained by the increase in the number of rumen microorganisms in the *Christensenellaceae_R_7_group*, which improves protein metabolism breakdown. This results in an increase in protein synthesis by rumen microorganisms and a consequent increase in the total amount of microbial protein in the small intestine. In turn, this increases the total amount of amino acids available for absorption and utilization in the small intestine, facilitating the improvement of yaks’ productive performance and thus increasing the protein content in yak muscle.

In conclusion, in our study, the rumen microbiome of yaks showed that increasing the dietary protein level increased the abundance and diversity of rumen bacteria. However, excessively high dietary protein levels were found to have a negative impact on the abundance and diversity of rumen bacteria. Yaks fed a diet with a protein level of 14% had a high feed conversion efficiency, and yaks fed a 12% protein level had better fat deposition and improved the quality and flavor of yak meat. Yak muscle metabolomics analysis further revealed that feeding a 12% protein diet may facilitate fat deposition in yaks, thus improving the quality and flavor of yak meat. Our findings not only provide new insights into the yak rumen microbiome at dietary protein level intake, but also characterize the yak muscle metabolome at different dietary protein level intakes and reveal correlations between yak rumen microbes and yak muscle metabolites. Characterization of the yak rumen microbiome and yak muscle metabolome may be useful in practice for determining dietary protein level intake.

## Data availability statement

The data that support the findings of this study are available from the corresponding author upon reasonable request, and the sequencing data are available from NCBI BioProject, PRJNA864640.

## Ethics statement

The animal study was approved by the Institutional Animal Care and Use Committee of Qinghai University. The study was conducted in accordance with the local legislation and institutional requirements.

## Author contributions

KP: Data curation, Writing – original draft, Writing – review and editing, JW: Investigation, Supervision, Writing – review and editing. SC: Data curation, Investigation, Project administration, Writing – review and editing. YY: Writing – review and editing. XW: Investigation, Software, Validation, Writing – review and editing. SL: Writing – review and editing. CD: Writing – review and editing. SW: Funding acquisition, Investigation, Project administration, Supervision, Writing – review and editing.

## Footnotes

1
http://www.ncbi.nlm.nih.gov/Traces/sra/

2
http://www.r-project.org/

3
http://www.metaboanalyst.ca/faces/ModuleView.xhtml

4
http://cran.r-project.org/web/packages/psych

5
https://cran.r-project.org/package=pheatmap
