# Supplementary material for: Ruminal microbiota and muscle metabolome characteristics of Tibetan plateau yaks fed different dietary protein levels
Source: Front Microbiol. 2024 Feb 14;15:1275865. doi: 10.3389/fmicb.2024.1275865 (PMC10899706; doi:10.3389/fmicb.2024.1275865)
Supplement: Supplementary file 1 [file Table_1.DOCX]

**Supplementary Table 1.** Effects of dietary with different protein levels on growth performance of Yak*.*

| Item^1^ | Groups^2^ | | | SEM^3^ | *P*-value |
| --- | --- | --- | --- | --- | --- |
|  | LP | MP | HP |  |  |
| Initial BW, kg | 199.80 | 200.65 | 200.30 | 2.57 | 0.298 |
| Final BW, kg | 265.00 | 280.60 | 268.00 | 2.01 | 0.173 |
| TWG, kg | 65.20 | 79.95 | 67.70 | 1.98 | 0.062 |
| ADG, kg/d | 0.72b | 0.89a | 0.75b | 0.02 | 0.027 |
| DMI, kg/d | 6.54a | 6.34ab | 6.21b | 0.04 | 0.002 |
| F/G | 9.03a | 7.14c | 8.26b | 0.10 | 0.033 |

^1^ BW body weight, TWG The total weight, ADG average daily gain, DMI dry matter intake. F/G = DMI/ADG. ^2^ LP: low dietary protein concentration group; MP: medium dietary protein concentration group; HP: high dietary protein concentration group.^3^ SEM, standard error of the mean.
